# More than Scales: Evidence for the Production and Exudation of Mucilage by the Peltate Trichomes of *Tillandsia cyanea* (Bromeliaceae: Tillandsioideae)

**DOI:** 10.3390/plants9060763

**Published:** 2020-06-18

**Authors:** Igor Ballego-Campos, Rafaela C. Forzza, Elder A. S. Paiva

**Affiliations:** 1 Departamento de Botânica, Instituto de Ciências Biológicas, Universidade Federal de Minas Gerais, Belo Horizonte, Minas Gerais 31270-901, Brazil; epaiva@icb.ufmg.br; 2 Jardim Botânico do Rio de Janeiro, Rio de Janeiro 22460-030, Brazil; rafaela@jbrj.gov.br

**Keywords:** scales, colleter, plant–environment interactions, plant secretion, gland, cuticular pores, desiccation, absorbing trichomes, herbivory

## Abstract

Bromeliad scales have been investigated extensively due to their recognition as a key ecological and evolutionary feature of Bromeliaceae. However, much remains unknown about such trichomes and only recently mucilage exudation was described for them in a species of the subfamily Bromelioideae. The present study aimed to investigate the secretion present in inflorescences of *Tillandsia cyanea* Linden ex K. Koch (Tillandsioideae) to determine whether the scales of this species also produce and release secretions. Samples of young and mature portions of inflorescences were collected and prepared according to standard methods for light and electron microscopy. Anatomical and ultrastructural results indicate that the secretion is produced by the wing portion of typical peltate trichomes on the adaxial surface of bracts. The secretory activity begins in the early stages of trichome expansion and characteristically occurs in cells exhibiting a porous cuticle and dense cytoplasm with numerous mitochondria and dictyosomes. Histochemical tests confirmed mucilage secretion and revealed proteins in the exudate. These data comprise the first record of mucilage exudation by trichomes within Tillandsioideae and indicate that this capacity may be more relevant to bromeliad biology than previously considered. Functional aspects and colleter-like activity are also discussed.

## 1. Introduction

The structure and function of the typical bromeliad indumentum have been studied for more than a century [1,2,3,4,5,6,7,8,9,10], and much work is still being performed in this regard [11,12,13,14,15,16]. In addition to being recognized as a synapomorphy of Bromeliaceae, the presence of peltate trichomes—traditionally referred to as “scales”—also represents an important ecological and evolutionary feature of the family. Accordingly, many functions have been previously described for these structures [8,9,11,16,17,18,19,20,21,22,23,24].

Amongst the eight subfamilies currently accepted for Bromeliaceae, Tillandsioideae is the most diverse with about 1486 species [25,26,27,28]. It is understandable, therefore, that trichomes in this subfamily have been extensively described in the literature, mainly due to their remarkably organized and complex structure, which is often associated with water and nutrient absorption capacity [2,7,8,9,15,18,21,29]. Such trichomes probably account for the evolution of extreme independence from the soil and, consequently, the acquisition of adaptive zones not exploited by any other epiphytes [18,30].

Despite growing knowledge about the function and structure of bromeliad scales, their secretory capacities are still mostly unknown. A glandular function was assigned to the leaf indumentum of *Tillandsia usneoides* (L.) L. due to the presence of an intracellular hydrophilic secretion accumulated in the uppermost cell of the trichome stalk [21]. Such secretory activity is potentially associated with an increase in water and nutrient absorption capacity [21]. However, as far as we know, no data have since been collected that expand knowledge about trichome secretion in Tillandsioideae.

Ballego-Campos and Paiva [16] recently demonstrated secretory activity associated with inflorescence trichomes of *Aechmea blanchetiana* (Baker) L. B.Sm. (Bromelioideae) and described a new glandular capacity related to the typical indumentum of the family. The scales of this species produce a mucilaginous secretion that covers young portions of the reproductive axis [16]. The functions associated with the secretion in *A. blanchetiana* appear to be the same as those attributed to the activity of colleters. These structures are currently characterized from a functional point of view as glands that produce and release mucilaginous or resinous secretion for protection against desiccation or herbivore/pathogen attack in young parts of the plant [16,31,32,33,34,35]. While colleters are widely distributed in eudicotyledons [36], their presence has been demonstrated in only a few monocot families [16,31,33,34,35,37,38,39]. 

Despite these recent advances, the knowledge gap regarding secretion in bromeliads is not restricted to the indumentum, and much remains to be understood about several aspects of secretory biology within Bromeliaceae [16,35]. Details on the origin and dynamics of secretions in this plant group are scarce [16,35,40,41,42], and many features concerning bromeliad exudates (e.g., fragrances, nectar, oils, gums, and mucilage) are not fully understood.

In this sense, we investigated the presence of mucilage observed in the inflorescences of *Tillandsia cyanea* Linden ex K. Koch, an epiphytic bromeliad that inhabits areas of tropical forest in Ecuador and Peru [43] and is widely known due to its extensive cultivation for ornamental purposes [44]. This investigation aimed to document the origin, nature, and dynamics of the secretion, as well as to test the hypothesis that inflorescence trichomes in Tillandsioideae can produce and release secretions.

## 2. Results

### 2.1. Secretory Activity and Aspects of the Secretion

**Figure 1:**
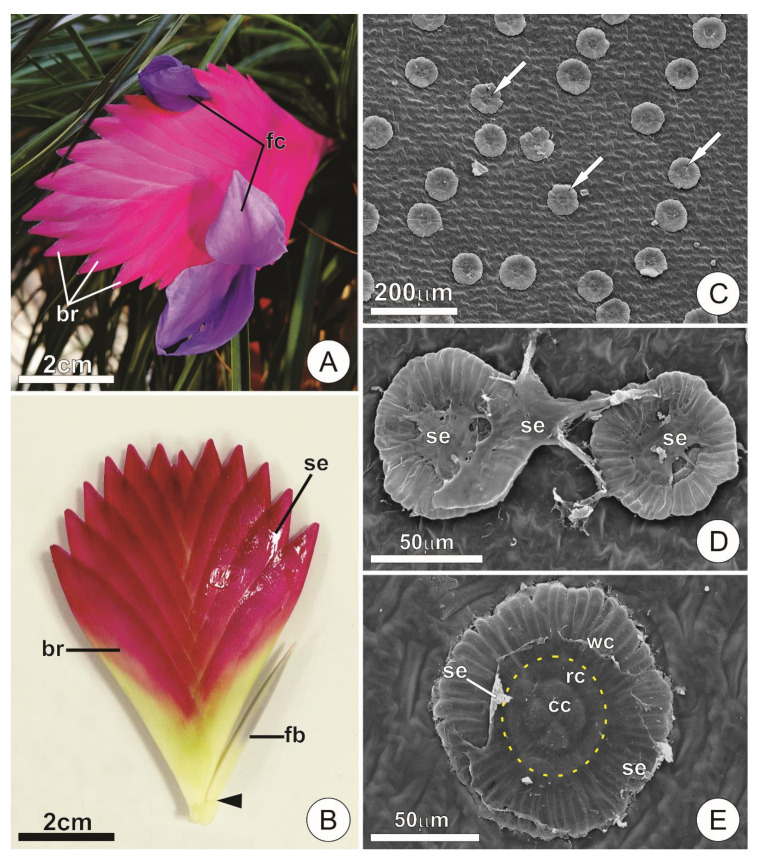
Distribution and aspect of secretory trichomes. (**A**,**B**) Overview of inflorescences at the flowering stage. Note the compact arrangement of bracts subtending the flowers with exposed corolla at anthesis (**A**) and the developing flower buds (**B**). The arrowhead indicates the removal of a bract. (**C**) Adaxial surface of a young bract showing numerous trichomes (arrows). (**D**,**E**) Detail of the secretory trichomes at different stages of expansion. Note the structure of the shield and a film of secretion covering its surface. The dotted area (in yellow) delimits the central disc. (br = bract; cc = central cells; fb = floral bud; fc = flower corolla; rc = ring cell; se = secretion; wc = wing cell).

The inflorescence of *T. cyanea* is entirely covered by large pink bracts associated with both the axis and the developing flower buds, which are arranged in an alternate distichous pattern along the main axis (Figure 1A,B). Flowers can be seen during anthesis, when only the corolla is exposed (Figure 1A).

The presence of secretion was observed throughout the adaxial surface of the bracts, usually accumulating in the space between them and covering the axis and flower buds. In natural conditions, the secretion was hyaline, viscous, odorless, and was not released to the outside of the spaces delimited by the bracts, such that it could only be observed on the surface of the inflorescences after manipulation (Figure 1B).

The secretion in mature bracts near the base of the inflorescence was relatively fluid and abundant, but was gradually more viscous and scarce towards the apex (i.e., young parts). When artificially exposed to the external environment, the secreted material drastically decreased in volume and turned into a solid film that was capable of rapid rehydration, recovering its volume and viscosity.

Secretory activity was detected only for the peltate trichomes covering the adaxial surface of young bracts. Such trichomes were usually seen coated by a film of the secretion (Figure 1C–E). Although present, trichomes on the abaxial surface of the bracts and main axis were sparse and did not present features that indicate a significant contribution to the exudate observed in the inflorescences.

### 2.2. Anatomy and Histochemistry of the Secretory Trichomes

**Figure 2:**
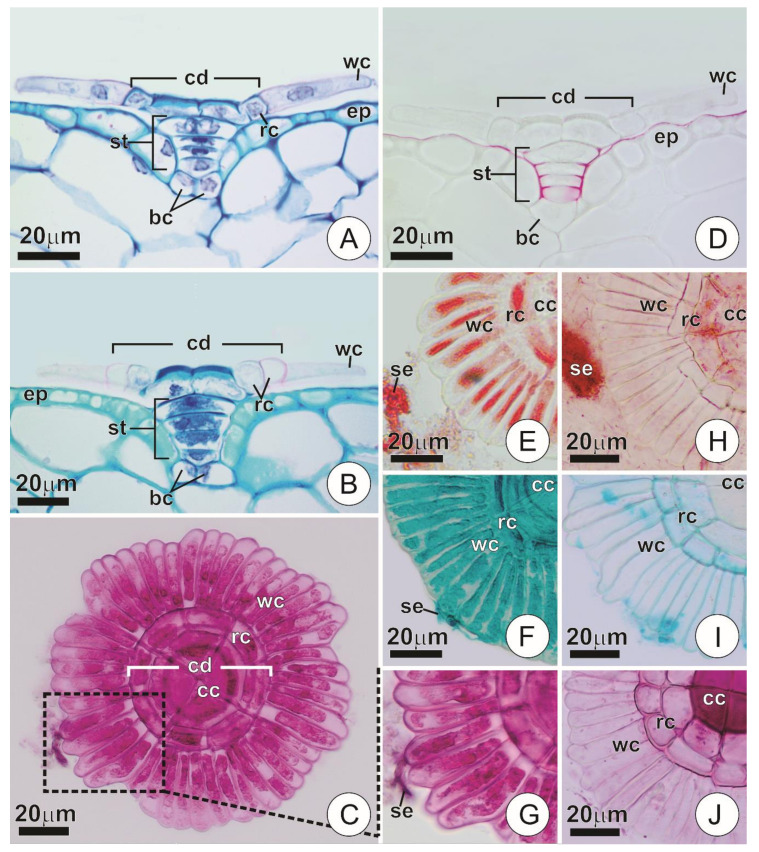
Anatomy and histochemistry of secretory trichomes. (**A**,**B**) Longitudinal section of a trichome showing the overall structure in young (**A**) and mature (**B**) bracts. Note the distinct appearance of the wing cells in each of the phases. (**C**) Shield of a secreting trichome. Note the overall structure and wing cells with conspicuous protoplasts marked for mucilage (in pink). (**D**) Longitudinal section of a trichome showing cuticle arrangement (in red). (**E**–**J**) Portions of the shield showing histochemical characterization of trichomes and secretion in young (**E**–**G**) and mature (**H**–**J**) bracts. (**E**,**H**) Xylidine pounceau, for proteins (in red). (**F**,**I**) Alcian blue, for mucilages (in blue). (**G**,**J**) Ruthenium red, for mucilages (in pink). (bc = basal cells; cc = central cells; cd = central disc; ep = ordinary epidermis cells; rc = ring cells; se = secretion; st = stalk; wc = wing cells).

Glandular trichomes of *T. cyanea* occur in depressions of the epidermis. Usually, they display a typical peltate pattern consisting of two basal cells, a short uniseriate stalk (3–4 cells) and a flat, squamiform apical portion that comprises the shield (Figure 2A–C). The uppermost cell of the stalk is often distinct in size and form, usually comprising a large, “dome” shaped cell (Figure 2A,B,D). In the shield, several cells are radially arranged forming two distinct portions: (a) a central disc consisting of four central cells usually surrounded by one or two rings of adjacent cells, and (b) a wing formed by a peripheral ring of elongated cells radially disposed around the central disc (Figure 1E and Figure 2A–C).

All cells comprising the trichomes of young bracts exhibited thin walls and somewhat dense protoplasts, which partially fill the cell lumen and may show conspicuous nuclei (Figure 2A). The cells of the wing, however, differed from the others as they exhibited especially thin pectic walls, particularly dense protoplasts and always distinct nuclei (Figure 2A,C). Such features were observed in trichomes at different degrees of expansion, even in those where the wings were close to their approximate total dimension (Figure 2A). The cuticle was detected covering the ordinary cells of the epidermis and extending along the anticlinal walls of the trichome stalk cells (Figure 2D). The cuticle also usually protruded through a small extension of the periclinal walls of the stalk cells, forming short flanges in that direction (Figure 2D). The presence of the cuticle was not noticeable in the remaining portions of the trichomes under light microscopy. Positive results for mucilages and proteins were obtained only in trichomes of young bracts, both for the protoplasts of the shield cells and for the exudate (Figure 2C,E–G). Tests regarding the other investigated substances showed negative results.

Mature bracts revealed structural changes exclusively in the cells that constitute the trichome shield, and especially in those comprising the wing, which lost their protoplasts and exhibited empty lumens at maturity (Figure 2B,H–J). The same process was observed in the cells that constitute the outermost ring of the central disc (when present). In contrast, the central cells and those comprising the inner ring usually kept their protoplasts in various degrees of vacuolation (Figure 2B,H). Also, the central cells in mature trichomes showed a remarkable thickening of their outer anticlinal walls (Figure 2B), with intense staining for pectin in tests with ruthenium red (Figure 2J). 

### 2.3. Ultrastructure

**Figure 3:**
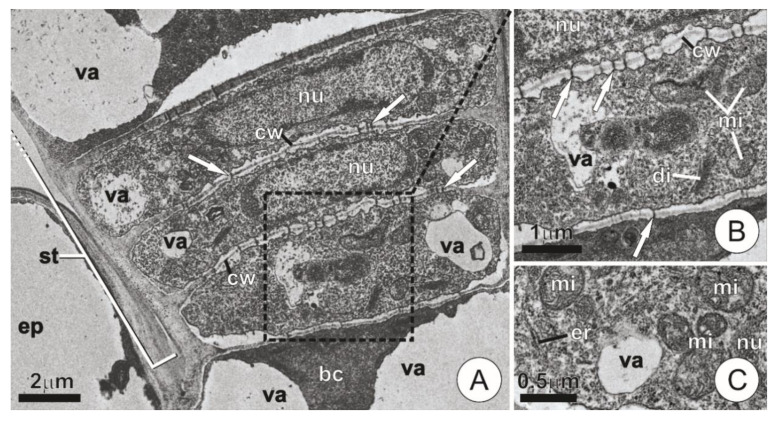
Ultrastructure of a trichome stalk and basal cells. (**A**,**B**) Longitudinal section showing overall aspect (**A**) and detail of the dotted area (**B**). Note the cells with thin walls largely connected by plasmodesmata (arrows). (**C**) Detail of a stalk cell showing dense, mitochondria-rich cytoplasm. (bc = basal cell; di = dictyosomes; ep = ordinary epidermis cell; er = endoplasmic reticulum; mi = mitochondria; nu = nucleus; st = stalk; va = vacuole).

Both the basal cells and those comprising the stalk of the secretory trichomes showed thin walls and formed an axial series largely connected by plasmodesmata (Figure 3A,B). Apart from the basal cells and the apical stalk cell—which usually displayed a well-developed vacuome—the remaining cells of the stalk generally exhibited dense cytoplasm and conspicuous nuclei (Figure 3A). Mitochondria were present in abundance, whereas dictyosomes occurred in lower numbers and did not show signs of intense activity (Figure 3B,C). Segments of endoplasmic reticulum, although present, were usually indistinct and viewed only as small fragments and vesicles dispersed throughout the cytoplasm (Figure 3C). There were no significant changes in the structural composition of the stalk cells among distinct phases of expansion. 

**Figure 4:**
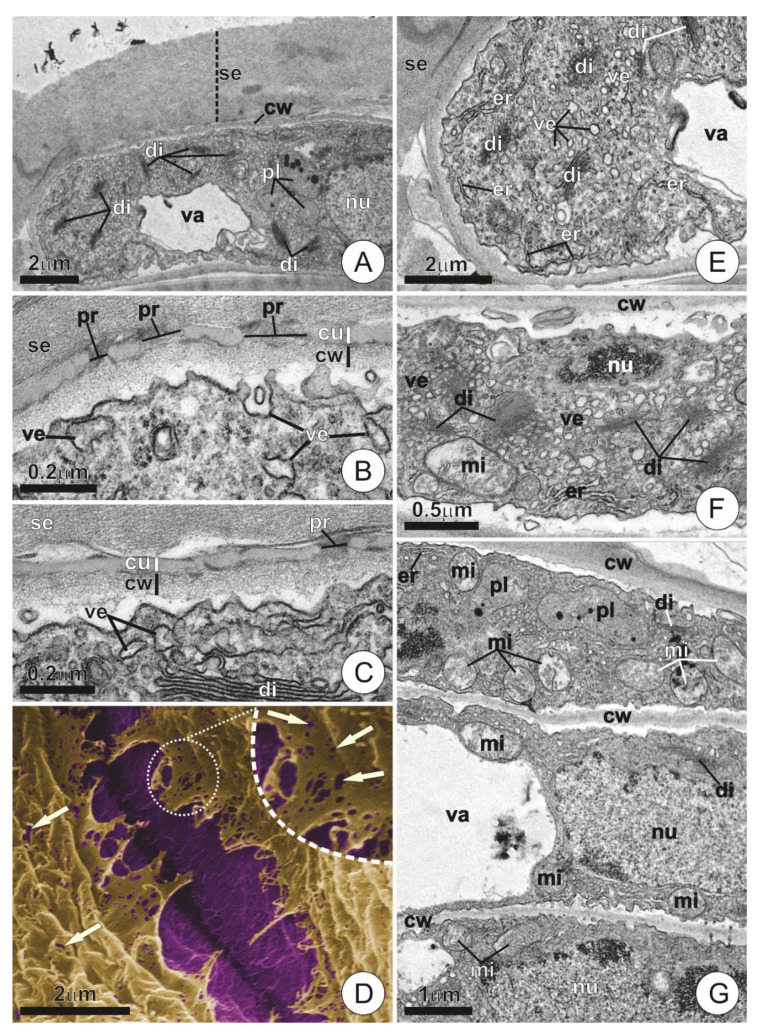
Ultrastructure of wing cells in young trichomes. (**A**) Longitudinal section of a wing cell. Note the dense cytoplasm and the thick layer of secretion on the cell surface. (**B**,**C**) Detail of the cell wall and protoplast. Note the presence of pores in the cuticle and small vesicles in contact with the plasmalemma. (**D**) Scanning electron micrograph showing the contact region between adjacent wing cells. Artificial coloring highlights the cell wall (purple) and the cuticle (ruptured, in yellow). Note the presence of cuticular pores (arrows). (**E**–**G**) Overview of wing cell protoplasts. Note the dense cytoplasm with numerous vesicles, dictyosomes, and mitochondria. (cu = cuticle; cw = cell wall; di = dictyosome; er = endoplasmic reticulum; mi = mitochondria; nu = nucleus; pl = plastids; pr = pore; se = secretion; va = vacuole; ve = vesicle.).

In the early stages of bract expansion, the wing portion of the trichomes usually presented cells with thin, inconspicuous walls, which were usually covered by a remarkably thin cuticle (c. 50 nm thickness) and a thick layer of secretion (Figure 4A–D).

Numerous pores of various diameters (c. 40–140 nm in diameter) were observed throughout the cuticle, both in cross-sections and in micromorphological analyses of the cuticular surface (Figure 4B–D). Wing cells at this stage also exhibited dense cytoplasm with highly developed Golgi apparatus usually composed of numerous active dictyosomes and vesicles dispersed throughout the cytoplasmic matrix (Figure 4A–C,E,F). Evidence of juxtaposition and fusion of these vesicles with the plasma membrane was observed (Figure 4B,C). The presence of mitochondria with well-developed cristae and segments of rough endoplasmic reticulum were especially noticeable, the latter usually appearing near the plasmalemma (Figure 4E–G). Plastids containing electron-dense bodies occurred only in small numbers and usually did not exhibit a well-developed inner membrane system (Figure 4A,G).

**Figure 5:**
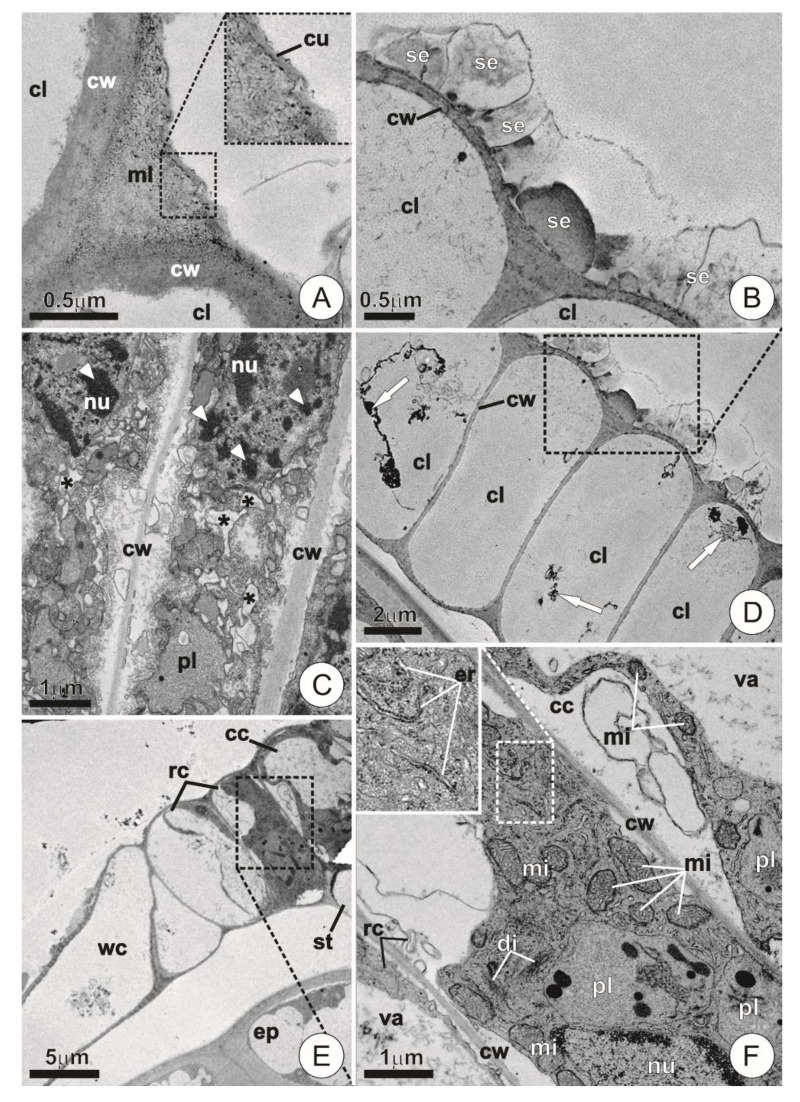
Ultrastructure of the wing and central disc cells in mature trichomes. (**A**) Detail of the region between adjacent cells showing conspicuous walls. Note the indistinct and discontinuous cuticle. (**B**) Detail showing secretion of fibrillar and compact aspect on the surface of mature wing cells. (**C**) Degenerating wing cells showing chromatin condensation/fragmentation (arrowheads) and the occurrence of vesiculation throughout the cytoplasm (asterisks). (**D**) Cross-section of the wing showing cells with empty lumens and residues of cytoplasm (arrows). (**E**) Overview of the shield in cross-section. Note the extravacuolar cytoplasm restricted to the periphery of the outer ring and central cells. (**F**) Detail of the dotted area in E showing protoplast composition. Note abundant mitochondria, plastids, and endoplasmic reticulum. (cc = central cell; cu = cuticle; cl = cell lumem; cw = cell wall; di = dictyosomes; ep = ordinary epidermis cell; er = endoplasmic reticulum; mi = mitochondria; ml = middle lamella; nu = nucleus; pl = plastids; rc = ring cell; se=secretion; st = stalk; va = vacuole; wc = wing cell.).

The wing underwent remarkable ultrastructural changes in the mature bracts. The cell walls in this portion became thicker and more defined, while the cuticle became largely discontinuous and indistinguishable (Figure 5A). Secretion was usually observed as compact and amorphous blocks or as fibrillar material located on the surface of the wing cells (Figure 5B). The composition of the protoplast was also distinct from that of young trichomes, especially due to the presence of cells showing protoplast degeneration (i.e., vesiculation, disintegration of membranes, condensation and fragmentation of chromatin; Figure 5C), or even empty lumens bearing nothing but small residues of cytoplasmic components (Figure 5D).

The cells that comprise the central disc of the trichomes, especially the central ones and those forming the inner ring, usually retained their protoplasts, exhibiting recognizable cytoplasm and organelles both in young and mature bracts (Figure 5E,F). Nonetheless, higher vacuolation in these cells usually caused the extravacuolar cytoplasm to be more or less restricted to the periphery of the lumen (Figure 5E,F). However, when larger portions of such extravacuolar components could be observed, they usually exhibited organelle-rich cytoplasm bearing numerous plastids, mitochondria, and segments of rough endoplasmic reticulum (Figure 5F). Dictyosomes were scarce and showed reduced activity in comparison with those of the wing cells of young trichomes (Figure 5F).

## 3. Discussion

### 3.1. Trichome Structure, Distribution, and Activity

Despite their secretory capacity, the structural features of the glandular trichomes of *T. cyanea* follow the peltate pattern typical of bromeliad scales, especially those of species of Tillandsioideae [7,8,18]. Thus, there seem to be no relevant structural differences between the glandular indumentum studied here and the scales typically covering the epidermis in various members of the Bromeliaceae. This observation is highly significant as it denotes a clear relationship of homology between these structures, even though they have potentially different functions.

Data on the structure of the studied trichomes suggest that the wing comprises a distinct portion due to both the general composition of its cells in young trichomes and to their loss of protoplasts in advanced stages of differentiation. In fact, as observed for this portion of the trichomes, the presence of dense protoplasts and conspicuous nuclei are common cellular features of secretory activity in plants, particularly when conditioned to the temporary occurrence. These secretory cell traits corroborate data obtained by several authors [16,35,36,45,46,47,48] and allow us to conclude that the wing constitutes the secretory portion of the trichomes.

In young bracts, typical secretory features observed in trichomes at different stages of expansion suggest that the secretory phase is simultaneous with trichome development and begins even during the initial stages of cell expansion and differentiation. The subsequent loss of protoplasts in the wing portion establishes that secretory activity takes place exclusively during the expansion of inflorescence parts (i.e., young bracts). The association between some plant secretions and juvenile organs is common [38,45,46,49,50] and was previously reported for the reproductive and vegetative axis of *A. blanchetiana* [16,35].

Histochemical tests confirm mucilaginous secretion and indicate the presence of proteins in the exudate. Secretions of this nature were observed previously in other similar secretory systems, including colleters in species of Orchidaceae [31,39] and several eudicotyledons [36,51,52]. The positive reaction provided by ruthenium red in the central portion of the shield indicates the presence of structural acid polysaccharides, rather than accumulated secretion inside cell compartments. This is especially likely since disc cells usually show an early increase in vacuolation and do not exhibit signs of secretion, as seen for the wing portion. Furthermore, we believe that the central disc comprises a structurally and functionally distinct portion that shares many affinities with the stalk cells, both in the composition of cell walls and in protoplast maintenance at maturity. This consideration will be further discussed in detail.

### 3.2. Ultrastructure and Secretory Mechanisms

Ultrastructural data support observations made at the structural level, reinforcing the intrinsic relationship of secretion to the wing of the trichomes, mainly due to the high cytoplasmic activity observed in this portion. The association between cytoplasm density and secretory activity is widely recognized and based on the increased abundance of mitochondria needed to provide the required energy while other organelles act in secretion production [53,54,55,56]. The presence of numerous active dictyosomes and segments of rough endoplasmic reticulum further confirms the secretion of polysaccharides and proteins [51,52,54], while the occurrence of vesicles close to or fusing with the plasmalemma indicates that secretion is released into the periplasmic space via a granulocrine pathway [54,55]. 

The presence of cuticular pores—which constitute true ducts by which secretion is released—is a unique record for typical bromeliad scales and an unusual occurrence even among other plant groups. Secretion release in plants generally requires (except for exudates released through the stomata) the accumulation of secretory products in the subcuticular space and subsequent exudation via cuticular ruptures [57]. In most known cases, hydrophilic pathways throughout the cuticle appear as channels of cell wall elements crossing the cuticle, rather than true ducts delimiting empty lumens [57,58,59]. Nevertheless, cuticle pores resembling those observed in *T. cyanea* have been described for, at least, nectaries of *Abutilon* [60] and *Spathodea campanulata* [57]. 

The fragmentation and loss of the cuticle observed in trichomes of mature bracts indicate that ruptures and detachments may occur throughout the secretory phase. Jeffree [61] suggested that, despite the occurrence of pores in the cuticle, ruptures could still play a role during the process of secretion release. However, considering that there are gaps in the original structure of the cuticle, its fragmentation may also be related to trichome expansion. In any case, the cuticle of the secretory trichomes of *T. cyanea* seems to constitute an inefficient barrier regarding the movement of hydrophilic secretions. Interestingly, the presence of a nonfunctional hydrophobic barrier was also reported for *A. blanchetiana*, although in this species cuticle inefficiency was associated with an apparent lack of lipid impregnation [16]. In mature leaf trichomes, the cutinized portion is usually restricted to the central disc, a feature that is likely associated with water and nutrient absorption capacity [2,4,7,8,20]. However, analyses of cuticle development in bromeliad scales are scarce, and water absorption capacity through the inflorescence needs confirmation.

In mature bracts, the presence of trichomes exhibiting degenerating protoplasts and cells with empty lumens corroborates the ephemeral nature of secretory activity. The occurrence of chromatin condensation, as well as darkening and fragmentation of the cytoplasm due to swelling of endoplasmic reticulum, were previously considered as evidence of programmed cell death in plants [13,62,63], and indicate that a comparable process operates on the secretory indumentum of *T. cyanea*. In this context, the occurrence of vesiculation throughout the cytoplasm can be interpreted as profuse dilations of the endoplasmic reticulum rather than due to vacuome development. These observations reinforce that cell death in the wing is a common process in bromeliad scales, both in the vegetative and in the reproductive axis [16]. The processes and mechanisms leading to this fate, however, remain poorly understood and need further research. In this sense, the scales of Bromeliaceae may be an interesting model for investigations concerning plant cell death.

The aspect of the material exuded on the surface of mature trichomes—namely the fibrillar and compact configuration of the secretion—can be explained by fluctuations in the viscosity of the mucilage in response to environmental conditions and the water status of the plant, especially during periods of low water availability and relative humidity. Such situations could potentially minimize water contact with the secretion, therefore affecting its overall aspect. Plant mucilages usually present hygroscopic features, exhibiting changes in their aspect and volume in response to the presence of moisture [16,45,47,64,65,66]. Furthermore, mucilaginous secretions displaying a fibrillar aspect were recorded in many similar secretory systems [16,35,46,67].

Ultrastructural data reinforce the functional distinction between the wing cells and those comprising the central disc, mainly due to the lack of numerous active dictyosomes. This feature indicates noninvolvement in polysaccharide secretion. Nevertheless, the presence of an otherwise dense extravacuolar cytoplasm in the central disc cells can be explained as a means of assuring control over potential water uptake capacity. The maintenance of “well-equipped” cytoplasm in the cells of the central disc, as well as stalk cells exhibiting dense, mitochondria-rich cytoplasm bearing indistinct segments of rough endoplasmic reticulum, were previously reported for the absorbing trichomes of leaves of *Tillandsia usneoides* L. [21]. According to this report, these features may be related to the passage of water and nutrients and controlled by plasmodesmata and the activity of an associated enzymatic pool [21]. 

Secretory activity in the inflorescences of *T. cyanea* is, in many ways, similar to that observed for *A. blanchetiana*, differing mainly in the organ that primarily bears the secretory indumentum and in some specific features of the glandular trichome (e.g., the structure of the cell wall and cuticle in the secretory phase). It is important to note that a glandular function associated with hydrophilic secretions was suggested previously for leaf trichomes of *Tillandsia usneoides* [21] and may as well occur in *Ananas comosus* (L.) Merr. [20]. However, in these species, secretion occurs as intracellular accumulation of hydrophilic material, and there is no apparent exudation outside of the plant. Thus, it constitutes an entirely different system from that described in the present work. Nevertheless, all these findings raise a point of great interest regarding the diversity and evolution of the secretory capacity of hydrophilic substances in bromeliad scales.

### 3.3. Functional Aspects

Many of the features presented by the secretory trichomes of *T. cyanea*, particularly the restriction of secretory activity to young portions and the mucilaginous nature of the exudate, allow their classification as colleters. This functional concept of colleter has been attributed to numerous secretory systems of this type, especially in the face of the recent descriptions of glands associated with juvenile organs in monocots [16,31,34,35,38,39]. Such glands seem to perform the same functions as traditional colleters, but usually lack the typical and relatively more complex structure often found in eudicotyledons [36]. 

In cases where the secretions of bromeliad inflorescences were studied in detail, their functional roles were attributed to both protection against desiccation of young parts [16] and defense against herbivory [68]. In the latter, although the glands accounting for the exudate were not described, the system appears to be similar to that of *T. cyanea*, and its secretory structures may presumably fall into the same functional category. Likewise, colleters were described in the vegetative axis of bromeliads, where they are also potentially involved in protection against desiccation [16]. Therefore, we suggest that the secretion present in the inflorescences of *T. cyanea* may act in both the protection and defense of young parts, especially during the development of flower buds and in the later expansion of the delicate corolla. In the latter, secretion may also contribute as a lubricant, facilitating the extrusion of the petals. 

A role in protection against desiccation is a recognized function of some exudates in young reproductive axes of angiosperms [31,34,38,39,52,69] in which water supply via xylem may be inefficient, and secretions could avoid water loss due to excessive transpiration or increase water uptake through the cuticle [45,47,69]. In this context, an additional role in water absorption must be considered concerning the trichomes of *T. cyanea* due to their similarity with the absorbing scales of bromeliad leaves, with which they share many structural features related to water uptake ability.

Moreover, while the mucilage in *T. cyanea* does not seem to provide effective immobilization of large insects and other herbivores, one cannot rule out potential protection against small arthropods and pathogens. In fact, the protein content found in the secretion constitutes a good piece of evidence in this direction, as the presence of proteins seems to be associated with defense against pathogen activity [70,71].

The retention of secretion in mature portions of the inflorescence (i.e., bracts of the basal portion, where secretory activity has already ceased) indicates that regardless of the presumable functions attributed to the exudate, they operate throughout the entire development of the reproductive axis, during flowering and, potentially, during fruit development as well. Such an observation suggests that the secretion may not be exclusively related to the protection of young organs, and instead act in a more complex and dynamic way. Future investigations regarding the functions of trichomes and their secretions in the inflorescences of bromeliads, especially related to water absorption capacity, may contribute to confirming these statements.

## 4. Materials and Methods 

### 4.1. Plant Material and Sampling

A total of three specimens kept in cultivation were sampled, two of which belong to Coleção de Plantas Vivas do Jardim Botânico da Fundação de Parques Municipais e Zoobotânica (Belo Horizonte, Brazil) and one acquired from commercial origin. Voucher specimens (BHZB 4713; BHZB 13215; BHZB 13216) were deposited in the BHZB herbarium of the same institution.

Samples of floral bracts (1 cm fragments of the median portion) and portions of the main axis (internodal fragments of ca. 3 mm each) were obtained, with the aid of a razor blade, from inflorescences in the early stage of flowering. Due to the acropetally maturation of the reproductive parts, portions in different stages of expansion were sampled from the same inflorescence according to the following categorization: (a) young portions (bracts and axis fragments at the distal third of the inflorescence, with about 50% or less of total expansion); and (b) mature portions (bracts or fragments of the axis in the middle and proximal third of the inflorescence with more than 50% of total expansion). 

Inflorescences were evaluated over two flowering episodes between the years 2018 and 2019. Whenever possible, more than one inflorescence was sampled per specimen. Bracts associated with the main axis of the inflorescence and those subtending the flowers were not distinguished here due to their similar structure and secretory capacity. 

### 4.2. Light Microscopy

Portions of bracts and of the inflorescence axis were subjected to a temporary vacuum before fixation for 24 h in a modified Karnovsky’s fixative (pH 7.2 in 0.1 M phosphate buffer, modified [72]). Fixed samples were then dehydrated in an increasing ethanol series and embedded in synthetic resin (2-hydroxyethyl-methacrylate, Leica^®^). Longitudinal and transverse sections (5–6 µm) were obtained using a rotary microtome (Hyrax M40, Carl Zeiss Mikroskopie, Jena, German) and stained with toluidine blue (pH 4.7, modified [73]), counterstained in an aqueous solution of ruthenium red (0.002%, m/v) and mounted in synthetic resin (Entellan^®^).

Histochemical tests were performed using fresh material to detect mucilages (ruthenium red [74] and alcian blue [75]), total lipids (sudan red 7B [76]), proteins (xylidine ponceau [77]), essential oils/oleoresins (NADI reagent [78]), alkaloids (Wagner’s reagent [79]), and starch (Lugol reagent [74]). In order to better visualize trichome histochemistry, samples of bracts were carefully scraped with a razor blade and the obtained material subjected to the tests mentioned above.

### 4.3. Electron Microscopy

Ultrastructural investigations applied scanning (SEM) and transmission (TEM) electron microscopy using samples of both young and mature bracts, which were subjected to temporary vacuum before fixation for 24 h in a modified Karnovsky’s fixative (pH 7.2 in 0.1 M phosphate buffer, modified, [72]). 

For SEM analysis, fixed samples were dehydrated in an increasing ethanol series, submitted to critical-point drying, and coated with a gold–palladium alloy. The prepared material was then analyzed using a Quanta 200 scanning electron microscope (FEI Company, Eindhoven, Netherlands).

For TEM analysis, fixed samples were postfixed in 1% osmium tetroxide (pH 7.2 in 0.1 M phosphate buffer) for 2 h, dehydrated in an increasing acetone series, and embedded in epoxy resin [80,81]. Ultrathin sections were obtained in an ultramicrotome (UC6, Leica Microsystems Inc., Deerfield, IL, USA) and contrasted using uranyl acetate [82] and lead citrate [83]. Sections were examined in a Tecnai G2–Spirit transmission electron microscope (Philips/FEI Company, Eindhoven, The Netherlands).

## 5. Conclusions

As far as we know, this is the first record for glandular activity by trichomes within Tillandsioideae associated with the release of a mucilaginous exudate. Such an account confirms that the secretory capacity of scales in bromeliad inflorescences is more common than previously considered and may constitute a feature with relevant ecological and evolutionary implications for Bromeliaceae. The presence of a porous cuticle in the early stages of trichome expansion also comprises a unique consideration in the development of the bromeliad indumentum, and the evidence for protoplast degeneration reinforces the occurrence of cell death in the typical trichomes of the family. Functional aspects associated with the secretory capacity of the trichomes of *T. cyanea* emphasize the importance of future investigations regarding biotic and abiotic interactions concerning the structure and function of the indumentum covering bromeliad inflorescences.
